# Waking up on the wrong side of the bed: sleep duration moderates the association between adolescent trait aggression and observed aggressive behaviour

**DOI:** 10.3389/fpsyg.2026.1705874

**Published:** 2026-03-10

**Authors:** Nicholas Denison, Michael Gradisar, Jessica Paterson, Serena Bauducco

**Affiliations:** 1 College of Education, Psychology and Social Work, Flinders University, Adelaide, SA, Australia; 2 WINK Sleep Pty Ltd., Adelaide, SA, Australia; 3 Sleep Cycle AB, Gothenburg, Sweden; 4 School of Behavioural, Social and Legal Sciences, Örebro University, Örebro, Sweden

**Keywords:** sleep duration, sleep quality, adolescents, aggression, controlled design

## Abstract

**Introduction:**

Prior research suggests that insufficient sleep can increase aggressive behaviour in adolescents. However, few studies have employed controlled designs, but none have incorporated objective measures of aggression. Moreover, the potential moderating role of sleep in the relationship between trait aggression and aggressive behaviour remains unexplored. This study addressed these gaps by examining whether sleep duration and quality moderate the association between trait aggression and objectively measured aggressive behaviour in adolescents. We hypothesised that adolescents higher in trait aggression would be more sensitive to poor sleep.

**Method:**

Thirty-four adolescent female participants spent one night in the Flinders University Sleep and Psychology Lab. Sleep duration was assessed using a consumer-grade sleep-tracking wearable, and sleep quality was self-reported. The following morning, participants underwent a noxious aggression provocation paradigm, after which behavioural aggression was assessed using a modified Hot Sauce Paradigm, measured via the weight of allocated wasabi paste. Trait aggression was measured using the Buss–Perry Aggression Questionnaire (BPAQ).

**Results:**

Sleep duration significantly moderated the relationship between trait aggression and aggressive behaviour, with shorter sleep predicting greater aggression among participants higher in trait aggression (*R*^2^_change_ = 0.11, *p* = 0.03). No such moderating effect was observed for self-reported sleep quality.

**Discussion:**

These findings suggest that adolescents high in trait aggression may be particularly susceptible to the behavioural consequences of shorter sleep. Ensuring adequate sleep could, therefore, be especially important for reducing aggression in this subgroup. This study extends prior research by demonstrating the moderating role of objectively measured sleep duration on aggression using a controlled design and a behavioural outcome measure, offering new insights for both theoretical models of aggression and the development of targeted, sleep-based interventions for at-risk youth.

## 1 Introduction

Human aggression, broadly defined, is a deliberate action intended to cause harm to another individual who is motivated to avoid that harm (Anderson and Bushman, 2002). Aggression may take many forms, such as verbal (e.g., shouting at someone), physical (e.g., hitting someone), or relational (e.g., giving someone the “silent” treatment) (Krahé, 2013). The motivation for aggression has been divided into two categories: hostile and instrumental (Anderson and Bushman, 2002). Hostile aggression is characterised as “hot,” impulsive behaviour, whereas instrumental aggression is premeditated and goal-oriented (Allen and Anderson, 2017); these subtypes are also referred to as “reactive” and “proactive” aggression (Parrott and Giancola, 2007; Dodge, 1991). The General Aggression Model (GAM; Anderson and Bushman, 2002) posits that several factors contribute to the processes leading to aggressive behaviour. These factors include an individual’s internal state (e.g., traits, mood, and arousal), situational factors (e.g., provocation, environment, and discomfort), the salience of the situation to the individual, and resource availability (e.g., cognitive capacity and time). In this study, we propose that healthy sleep may be a valuable resource to reduce the likelihood of aggressive acts—whereby either sleep duration, sleep quality, or both may moderate the association between a person’s existing level of trait aggression and their tendency towards aggressive behaviour.

Trait aggression can be defined as an individual’s susceptibility to perceiving non-threatening or ambiguous situations as hostile, along with the propensity to attribute hostile intent to the actions of others (Crick and Dodge, 1994; Dill et al., 1997). Just as behavioural aggression can be expressed in many forms, trait aggression can similarly comprise multiple components, including physical aggression, verbal aggression, anger, and hostility (Buss and Perry, 1992). Logically, higher levels of trait aggression should relate to a greater probability of acting aggressively, and this has been confirmed in both neutral settings and situations in which the individual is provoked (Bettencourt et al., 2006). Indeed, situational provocation has been theorised as necessary for observing aggressive acts when a key resource—that is, sleep—is depleted (Krizan and Herlache, 2016). Finally, it has been suggested that children and adolescents represent a particularly relevant population in which the effects of sleep-affected behavioural aggression can be studied, given that peer-to-peer bullying is both prevalent and costly (Krizan and Herlache, 2016).

Aggressive behaviour increases during adolescence (Lynne-Landsman et al., 2011). Coinciding with the onset of puberty is a combination of individual biological and environmental factors that place adolescents in a vulnerable position regarding emotional coping resources (Valois et al., 2002) and the regulation of negative emotions (e.g., anger; Cracco et al., 2017), which in turn increases the possibility of aggressive behaviour (Roberton et al., 2012; Purwadi et al., 2020). The onset of puberty also coincides with significant changes in adolescents’ bioregulatory control of sleep, including a reduction in sleep homeostatic pressure and a delay in the 24-h circadian rhythm (Crowley et al., 2018; Campbell et al., 2024), resulting in a delayed onset of sleep. Thus, as adolescents age, they experience a gradual restriction of sleep duration on school nights (Gradisar et al., 2011b; Gariepy et al., 2020). Whereas sleep duration reflects the total time spent sleeping, sleep quality is a self-reported measure, often reflecting the subjective experience of sleep, and the degree to which an individual feels refreshed upon waking (Dewald et al., 2010). Both sleep duration and quality have demonstrated robust relationships with an array of negative outcomes (e.g., physical and mental health, and academic performance; Dewald et al., 2010; Shochat et al., 2014; Short et al., 2020), and similar relationships have been observed in studies examining sleep and aggression.

Meta-analyses have identified a relationship between aggression and sleep duration (*r* = −0.16; Van Veen et al., 2022) and between aggression and sleep quality (*r* = 0.38; Van Veen et al., 2021). However, the majority of included studies in both cases were correlational in nature, which not only raises questions regarding causality but also overlooks the processes leading to aggressive behaviour. Furthermore, some studies included in these meta-analyses examined mood states (e.g., anger) rather than behavioural aggression (Baum et al., 2014; Short and Louca, 2015; Van Dyk et al., 2017). Previous studies have generally conceptualised sleep as a causal factor in aggression (see Van Veen et al., 2021; Van Veen et al., 2022; Kamphuis et al., 2012), whereas we are unaware of investigations that have examined sleep as a moderating factor of the association between trait aggression and aggressive behaviour. That is, no studies have probed whether adolescents high in trait aggression are more predisposed to exhibiting aggressive behaviour following reduced sleep duration and/or poor sleep quality compared to those lower in trait aggression. The rationale for this enquiry is that the cognitive resources affected by sleep (Lo et al., 2016; Dewald et al., 2010; Short et al., 2020)—particularly those related to situational reappraisal (Anderson and Bushman, 2002)—may hold greater significance for individuals higher in trait aggression, as they are more predisposed towards making hostile attributions in non-threatening scenarios and towards others (Crick and Dodge, 1994; Dill et al., 1997). In other words, in the face of poor sleep, some individuals may lack the cognitive capacity to fully evaluate a situation and may therefore respond more impulsively.

The current study, therefore, aims to fill the current gap in our knowledge regarding objectively measured reactive aggression in relation to trait aggression, as moderated by sleep duration, in a controlled setting. As sleep quality has also been identified as an important factor alongside duration (see Van Veen et al., 2021; Dewald et al., 2010), and no known controlled laboratory studies have included indices of both, its moderating role will also be investigated in the present study.

## 2 Methods

This study was part of a larger laboratory investigation examining female friendships, technology use, and sleep. Girls were selected as the majority of experimental studies on the association between technology and sleep have largely focused on boys and video gaming, whereas girls, who generally prefer social media use (Smith et al., 2020), have been under-represented (e.g., Ivarsson et al., 2013; Reynolds et al., 2015; Smith et al., 2017). Over two nights, participants were exposed to a “social night” and a “non-social” night. While participants’ sleep duration was not directly manipulated, we expected a spread of sleep durations and a higher likelihood of short sleep duration when girls socialised with each other (i.e., “social night”). Thus, the current study utilised data from the “social night” condition to investigate the moderating effect of sleep duration on the relationship between trait aggression and aggressive behaviour.

### 2.1 Participants

A total of 34 girls (mean age = 17.1 years, SD = 0.7) were recruited online (e.g., Facebook, Instagram, and TikTok) and through physical flyers within the community. The inclusion criteria were (i) identifying as female; (ii) being 16–18 years of age; (iii) willingness to participate together with a close friend, assessed using the McGill Friendship Questionnaire (Mendelson and Aboud, 1999); (iv) absence of severe mental health symptoms, as measured by the Depression Anxiety and Stress Scale (DASS-21) (Lovibond and Lovibond, 1995); (v) no indication of a sleep disorder, defined as self-reported sleep problems more than three to four times per week, napping more than five times per week, or late weekday bedtimes [after 12:30 a.m.] and wake times [after 9:00 a.m.]; (vi) not using medications known to affect sleep (e.g., melatonin); and (vii) not currently smoking or vaping. Of 80 intake screenings, 18 potential participants met exclusion criteria and 17 were lost after screening. No participants withdrew after commencing the study. Written informed consent was obtained from all participants and from the parents of adolescents under 18 years of age. Participants received an AUD$30 gift voucher for each night of participation. This study was approved by the Flinders University Social and Behavioural Research Ethics Committee. The experiment was not preregistered.

### 2.2 Measures

#### 2.2.1 Trait aggression

The Buss-Perry Aggression Questionnaire (BPAQ) is a 29-item self-report measure of trait aggression (Buss and Perry, 1992). Each item is scored on a 5-point Likert scale. The measure comprises four subscales: verbal aggression (five items), physical aggression (nine items), anger (seven items), and hostility (eight items). Subscale scores are combined to form a composite overall score of trait aggression. The BPAQ has reportedly good construct validity (Archer and Webb, 2006) and reliability (Buss and Perry, 1992). The internal consistency (Cronbach’s *α*) values for the BPAQ subscales in this study were as follows: physical (*α* = 0.715), verbal (*α* = 0.659), anger (*α* = 0.818), and hostility (*α* = 0.730). Across all 29 items, the BPAQ demonstrated a Cronbach’s *α* of 0.857.

#### 2.2.2 Behavioural aggression (wasabi task)

This measure is an adaptation of Lieberman et al.'s (1999) Hot Sauce Task, which assesses aggression by asking participants to allocate wasabi for another person to consume (Sauer et al., 2015). The amount of wasabi allocated serves as an index of aggression as it imposes an unpleasant experience on an ostensibly real individual. The task’s effectiveness relies on participants understanding that (a) the wasabi is very hot, (b) consuming wasabi on its own is generally unpleasant, and (c) the other person will consume the entire allocation, as per the cover story provided.

As a cover story, participants were told that a study concerning tasting experiences was being conducted nearby and that the researchers required tasting samples. They were instructed to allocate wasabi for this study so that the researchers could remain blind to the serving size. Participants were informed that they could put as much or as little wasabi into a cup for the other person as they wished, and that the other study’s participants had agreed to consume the entire portion allocated. Non-transparent cups with lids were provided, and participants were left in private to make their allocations.

To improve the believability of the cover story, we included food-related elements in the study’s name (“The Sleep Eat Chat Study”), food-themed questions in the screening, and a mock food-tasting questionnaire on the social night (i.e., the night preceding the wasabi test).

This wasabi measure has been used effectively to detect aggression in studies related to video gaming (Sauer et al., 2015; Drummond et al., 2021), violent musical lyrics (Mast and McAndrew, 2011), and social rejection (DeWall et al., 2013). It demonstrates ecological validity, given real-world instances of food being spiked with excessive spice (Lieberman et al., 1999), and convergent validity, with a moderate correlation to the BPAQ composite score (*r* = 0.30; Lieberman et al., 1999).

#### 2.2.3 Sleep

##### 2.2.3.1 Sleep duration

Total sleep time (TST), a measure of the time between sleep onset and waking, not accounting for wake after sleep onset (WASO), was measured using a Polar Unite sleep-tracking watch. Recent studies (e.g., Miller et al., 2022; Pesonen and Kuula, 2018; Stone et al., 2020; Chinoy et al., 2021) have validated the accuracy of modern sleep-tracking watches, identifying them as “promising alternatives to research-grade actigraphy” for measuring the time window between sleep onset and sleep offset (Chinoy et al., 2021, p. 514). A Polar model, similar to that used in this study (Polar A370), was found to have a mean difference of only 5 min for sleep onset and less than a minute for sleep offset (waking) when compared to polysomnography (PSG) in a study validating the watch on adolescents (Pesonen and Kuula, 2018).

##### 2.2.3.2 Sleep quality

Sleep quality was measured through two 10-point Likert scales completed by participants in the morning. One asked about the participant’s subjective assessment of their sleep experience, asking: “How would you rate the quality of your sleep last night?”(*1* = *Poor, 10 = Very Good*). The other assessed how refreshed the participant felt, asking: “*How are you feeling this morning?*” (*1 = Tired, 10 = Refreshed*). The two scores were summed to create a composite score.

### 2.3 Procedure

Participants attended the sleep laboratory a week prior to their laboratory stay to complete baseline measures (BPAQ, wasabi, mock food-related questions, and the parallel studies’ measures), familiarise themselves with the laboratory, and provide written consent. Participants who had never tried wasabi paste were offered a sample to try at this session, as the baseline questionnaire included items on their typical wasabi consumption. Participants then scheduled their two laboratory nights and were subsequently randomised to the “alone night”/“social night” conditions.

Prior to participant arrival on the evening of the social night condition, researchers concealed alarm clocks under participants’ beds and set the main room lights to full brightness for the wake-up provocation. The externally controlled main room lights were then turned off and replaced by soft bedside lamp lighting. After arriving at the laboratory in the evening (6 p.m.), the participants had dinner together; were fitted with their sleep-tracking watches; and answered the mock food-tasting questionnaire, an online questionnaire that included the DASS-21 stress subscale, and other measures for the parallel studies. Prior to retiring to their bedrooms, participants were briefed on their instructions for the night. Participants were asked not to set any alarms for the morning as they would be awoken at 6:30 a.m. by the researchers. After this briefing, participants were asked if they minded doing the researchers a favour in the morning, namely, allocating the wasabi paste for the fictional other study (wasabi task). Participants were told they would perform this task first thing after waking, as close as possible to the provocative alarm and bright light, in order to capture their reactive response. Participants then retired to their individual bedrooms at 8 p.m., chatting with their friend via WhatsApp and watching Netflix until they decided to sleep.

**Figure 1:**
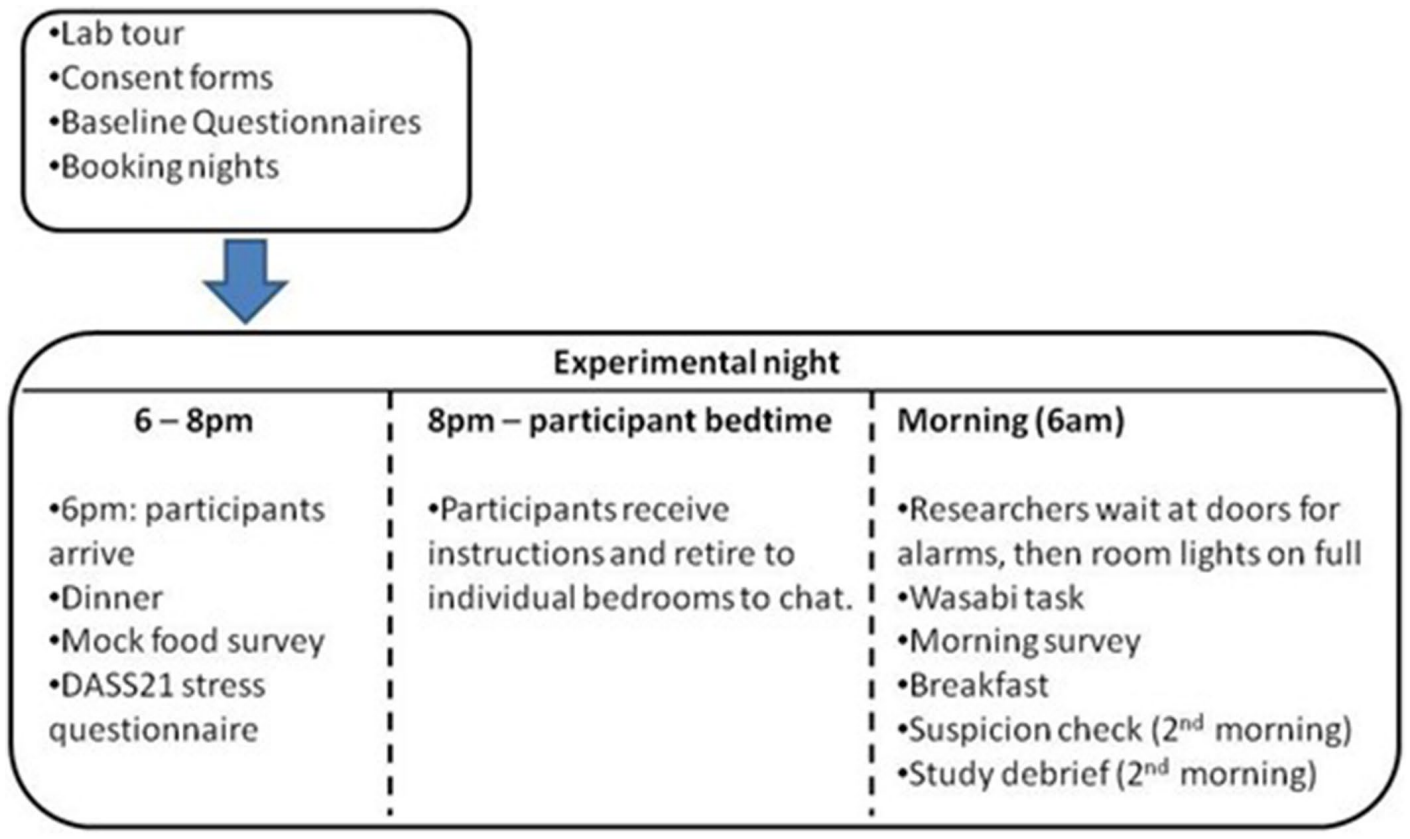
Experimental night procedure.

In the morning, researchers waited at the bedroom doors until 6:30 a.m. Upon hearing the alarms, the researchers turned on the bedroom lights and handed participants the wasabi task materials. Following the wasabi task, participants completed an online questionnaire containing measures of self-reported sleep quality. Participants completed a suspicion check on their final morning, prior to debriefing, using a 10-point Likert scale, assessing their belief in the fictional cover story (i.e., relating to the wasabi allocation rationale) from 1 = not at all believable to 10 = very believable. After debriefing the participants and their subsequent departure, the wasabi allocations were weighed (in grams) and recorded. As a manipulation check, annoyance at the waking provocation was assessed with a 10-mm VAS, which asked: “Were you annoyed at being woken up by the bright light and alarm this morning?” An open-ended question asked participants to explain any known motivations for allocating the wasabi amount they did. Responses were coded from most empathetic to aggressive, creating an ordinal variable (see Table 3). Figure 1 visually details the procedure.

### 2.4 Statistical analyses

Prior to analysis, data were screened for outliers and regression assumptions. Three high-end outliers in the wasabi allocation data were identified and winsorised following standard recommendations (Tabachnick and Fidell, 2007). Despite this, two cases showed excessive influence in the regression models (Cook’s distance values exceeding 4/n and problematic residual and P–P plots; Tabachnick and Fidell, 2007). As the wasabi allocation data were positively skewed, a square root transformation was undertaken to improve its suitability for regression. Positively skewed data are common in “hot sauce paradigm” studies (Lieberman et al., 1999), and the square root transformation is often used (Ayduk et al., 2008; DeBono and Muraven, 2014). After transformation, the high Cook’s distance cases remained and were therefore excluded from analysis, as recommended by Tabachnick and Fidell (2007). Sensitivity analyses excluding influential cases indicated no meaningful changes in the pattern or significance of the results. Statistical analyses were conducted using SPSS version 28.0.1.0. Moderated regressions were undertaken using Hayes (2017) PROCESS macro, Version 4.1 (Model 1). Prior to testing the study’s hypotheses, preliminary analyses were undertaken to assess any confounding issues. An independent samples *t*-test assessed mean wasabi allocation for those who undertook the present study on their first laboratory night (*n* = 15, *M* = 0.96, SD = 0.59) compared to their second night (*n* = 17, *M* = 1.16, SD = 0.80), finding no significant difference, *t*(30) = −0.82, *p* > 0.05.

## 3 Results

Table 1 presents the descriptive statistics of the study. Objectively-measured sleep averaged below the recommended 8–10 h per night (Hirshkowitz et al., 2015), with 21.9% (*n* = 7) sleeping less than 7 h. Average stress on the social night was in the normal range (<15) (Lovibond and Lovibond, 1995). The manipulation check revealed only a moderate annoyance with the wake-up procedure, and the suspicion check suggests that the cover story was generally well accepted.

**Table 1:** Means and standard deviations for main study variables.

Variable (range)	*M*	SD
Wasabi (0.06–9.33)	1.69	2.4
Sleep duration (5.15–8.72)	7.44	0.9
Sleep quality (3–10)	6.47	2.0
Sleep refreshment (2–10)	5.56	2.1
Stress (1–15)	7.63	4.1
Manipulation check (0.7–9)	3.45	2.5
Suspicion check (2–10)	6.89	2.5
Wasabi preference (0.00–3.25)	0.47	0.9
Wasabi liking (1–5)	2.47	1.4
BPAQ comp (49–111)	73.72	13.65

*N* = 32. Wasabi, wasabi allocation in grams; Wasabi preference, self-reported typical serving preference for wasabi; Wasabi liking, self-reported liking of wasabi taste; Sleep, sleep total in hours; BPAQ comp, BPAQ composite (i.e., total).

Regarding the scores for the BPAQ, compared with Buss and Perry’s (1992) original validation scores for female participants, the present study’s scores are marginally higher, except for the physical aggression subscale mean, which is identical. Supplementary Table S1 shows means and standard deviations for the BPAQ subscales and total score.

To test whether sleep duration moderates the relationship between trait aggression and aggressive behaviour, a moderated regression was performed with the transformed wasabi allocation as the dependent variable, and trait aggression and sleep duration were used as the predictor and moderator, respectively. The potentially confounding variables of liking of wasabi, participant’s typical serving size of wasabi, stress, and cover story suspicion were entered as covariates and controlled for in the analysis. The analysis revealed a significant interaction, *R*^2^_change_ = 0.11, *p* = 0.03. Together, the variables accounted for 49% of the explained variance in aggressive behaviour (wasabi allocation). Table 2 presents the unstandardised coefficients associated with the regression.

**Table 2:** Regression coefficients from a test of the moderating effect of sleep on the trait aggression aggressive behaviour relationship.

Variable	*b*	SE*_b_*	95% CI for *b*	*p*
Constant	0.73	0.58	[−0.47, 1.91]	0.22
BPAQ comp	−0.003	0.009	[−0.02, 0.02]	0.76
Sleep duration	−0.007	0.002	[−0.01, −0.002]	0.01
Wasabi preference	−0.018	0.15	[−0.33, 0.29]	0.91
Wasabi liking	0.10	0.11	[−0.12, 0.32]	0.34
Stress	0.006	0.03	[−0.06, 0.08]	0.85
Suspicion check	0.003	0.05	[−0.11, 0.11]	0.96
BPAQ comp * Sleep duration	−0.0003	0.0001	[−0.0005, 0.0000]	0.03

bs are unstandardised regression coefficients. All *p*-values are based on *t*-tests with df = 24. BPAQ comp and sleep duration were centred for the analysis.

**Figure 2:**
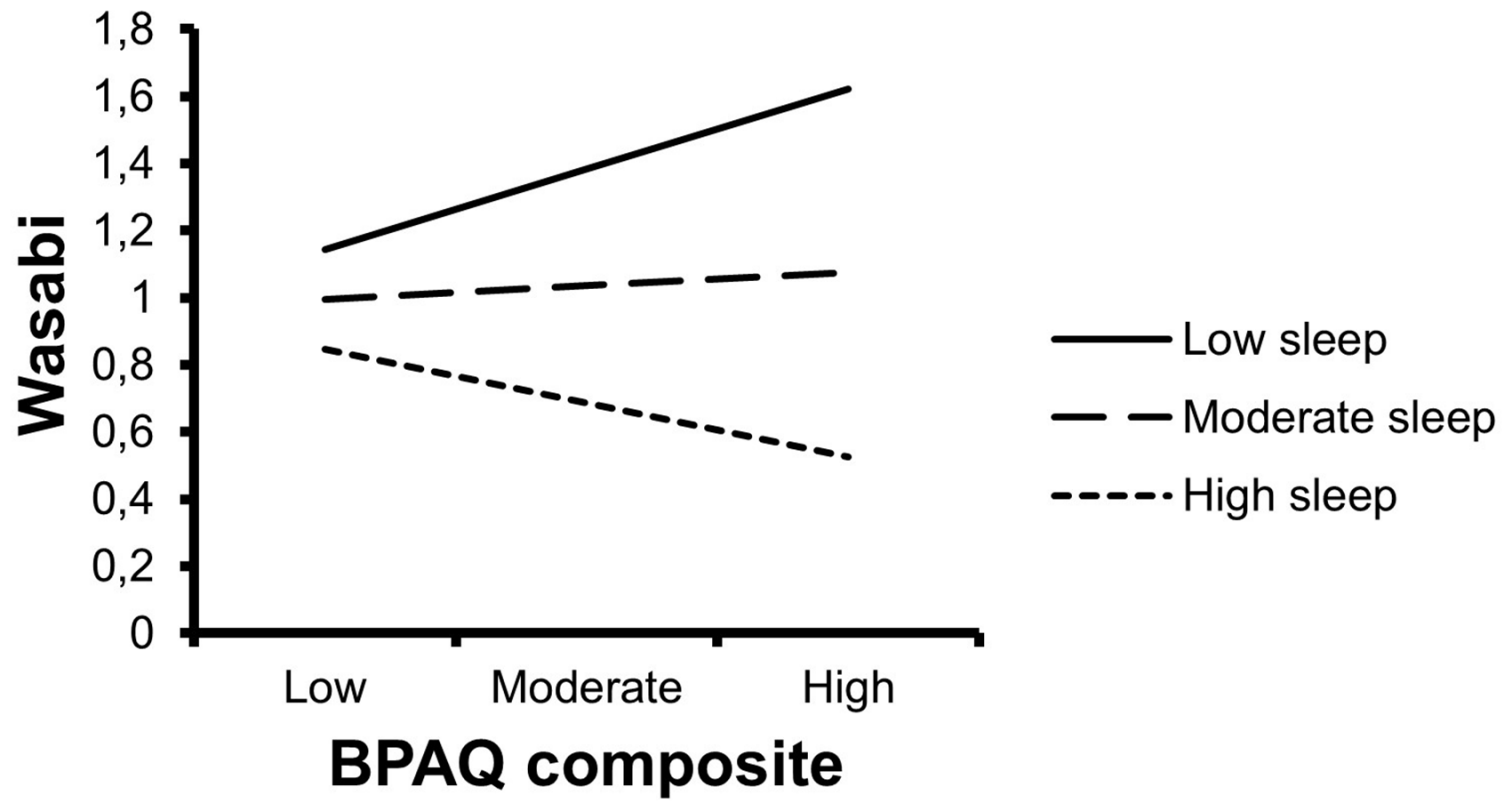
The moderating effect of sleep upon trait aggression and behavioural aggression. High and low moderator lines represent 1 standard deviation (52.25) above and below the mean, respectively.

The interaction suggests that the effect of trait aggression on aggressive behaviour varies with sleep duration. Specifically, individuals low in trait aggression were not as affected by sleep duration as those higher on the spectrum of trait aggression. Figure 2 indicates that longer sleep duration is associated with higher trait aggression to respond less aggressively than those with lower trait aggression. The mean sleep duration (approximately 7.5 h), shows a comparable effect across low, moderate and high aggression.

A moderated regression was conducted with the sleep quality composite score as the predictor, along with the same covariates and dependent variables as in the previous analyses. This interaction proved to be non-significant, *R*^2^_change_ < 0.01, *p* = 0.72.

The qualitative open-ended responses were analysed for themes. Participants were asked why they allocated the wasabi amount they did. Themes of empathy, neutrality/vagueness, veiled aggression, and overt aggression were identified and coded. These themes were scored on a range of 1–4, producing an ordinal scale from empathetic to aggressive. Two scorers coded the responses independently, and 75% were in agreement (*n* = 24 of 32). To ensure an unbiased scoring, scores from the researcher, independent of the study, were used in the analysis. Table 3 provides example quotes from the response set.

**Table 3:** Open question response coding and example quotes.

Coding	Example quote
1. (Empathetic)	“I know too much wasabi is painful. I did not want them to suffer.”
2. (Neutral or vague)	“I wanted it to look pretty to eat, so I did a swirl.”
3. (Possible undertones of aggression)	“I feel like that is a regular amount for people who like wasabi? Also, it’s not me who has to try it LOL.”
4. (Candidly aggressive)	“I’m tired and not really aware of what is going on, so I decided, why not – and my sleep was pretty bad, so if I suffer, so do they.”

In order to investigate potential relationships between the ordinal open question responses (Table 3) and the other study variables, non-parametric Spearman’s rank correlations were performed with the ordinal data and variables of interest. A large positive correlation was found with wasabi allocation, *rs*(30) = 0.72, *p* < 0.001, and a small-to-moderate negative correlation was found with the sleep quality (composite), *rs*(30) = −0.37, *p* = 0.04, suggesting that more aggressive responses were associated with larger wasabi amounts and worse sleep quality. No significant relationships were found with the BPAQ composite scale (or subscales), sleep duration, suspicion check, manipulation check, or stress.

## 4 Discussion

This study set out to investigate sleep as a moderator in the relationship between adolescent trait aggression and aggressive behaviour. It was predicted that sleep would act as a moderator, whereby those higher in trait aggression would be more affected by worse sleep duration in displaying reactive aggression. The findings of the present study lend support to this hypothesis and also contribute further evidence to the broader literature investigating sleep duration as a resource influencing aggressive behaviour (Van Veen et al., 2022; Kamphuis et al., 2012). We also investigated self-reported sleep quality as a variable in relation to aggression but found no relationship between sleep quality and aggression, which is in opposition to previous studies (Van Veen et al., 2021).

Adolescents with high trait aggression were more affected by sleep duration compared to those with low trait aggression. It is worth noting that, at approximately 7.5 h (see Figure 2), sleep had little interactive effect across the trait aggression spectrum. Conversely, at 1 h less (6.5 h) and 1 h more (8.5 h), those higher in trait aggression responded more aggressively and less aggressively, respectively. These findings align somewhat with the demarcations of recommended sleep duration (<7 h) for adolescents (Hirshkowitz et al., 2015; Leger et al., 2012) and the recommended range for healthy sleep (8–10 h) (Hirshkowitz et al., 2015). The main practical implication resulting from our study findings is to protect sleep quantity via earlier bedtimes. Maintaining healthy bedtimes is one of the most effective ways to ensure sufficient sleep duration in adolescents, particularly because wake-up times are often externally constrained—a pattern we intentionally simulated in our experimental design. Potential approaches to instill healthy bedtimes span multiple levels, including individual interventions (e.g., CBT-I, Galgut et al., 2025; bright light therapy, Gradisar et al., 2011a), family-based strategies (e.g., parent-set bedtimes; Bauducco et al., 2024), and school-level initiatives (e.g., Bonnar et al., 2015) aimed at supporting regular sleep schedules and promoting consistent, healthy bedtime routines.

When considered in the context of Anderson and Bushman’s (2002) General Aggression Model (GAM), sleep appears to function as a resource at the automatic appraisal stage, moderating the effect of the internal state (e.g., trait aggression). As those higher in trait aggression are more likely to perceive ambiguous situations as hostile and also more readily attribute hostile intent to others’ actions (Crick and Dodge, 1994; Dill et al., 1997), cognitive resources, provided by sleep, appear necessary to override impulsive reactions. Some insight into the appraisal process can be gleaned from participants’ qualitative responses. There appeared evidence not only of effortful appraisal, particularly in empathetic responses (e.g., “…I thought, what if I had to eat it?”), but also malicious responses (e.g., “…when I heard they would eat it all, I wanted to give them the amount I did”). Evidence of impulsive responses can also be seen, “…so I decided, why not—and my sleep was pretty bad, so if I suffer, so do they.” It should be noted that participants answered these questions after the fact; therefore, their responses represent an attempt to understand their own decision-making processes but not the content of the process itself.

While the present study posits sleep as an important resource within the General Aggression Model (GAM), sleep may be viewed as both a cognitive and emotional resource. Sleep restriction studies have shown broad-reaching impairments on performance measures that are underpinned by cognitive processes not too dissimilar to those proposed in the GAM (i.e., time, cognitive capacity; Allen and Anderson, 2017). From an emotional perspective, anger is the second-highest correlate with low sleep duration, after positive mood (Short et al., 2020). The GAM proposes that anger is derived from the fight-side of sympathetic nervous system activation, when triggered by an aversive event (Allen and Anderson, 2017). Thus, lowered sleep duration in vulnerable adolescents (i.e., high trait aggression) may not provide the combined cognitive and emotional restraints necessary to automatically appraise and reappraise events, ultimately increasing the odds of an aggressive act. The present study used an aversive event, common to many older adolescents (onset of loud alarm + bright light stimulus), yet adolescents’ mean provocation rating was below the midpoint (3.9 out of 9). Given that our data support the processes outlined in the GAM, it may be that our self-reported provocation ratings were influenced by self-reported biases (e.g., social desirability). Alternatively, rather than the waking provocation, the immediate situational factor for reactive aggression may have been the wasabi task itself (i.e., annoyance at being subjected to doing such a task at 6:30 a.m.).

The interaction between trait aggression and sleep duration also produced a surprising effect; specifically, adolescents with high trait aggression responded less aggressively than their low trait peers—when sleep duration was high. As adolescents are believed to be challenged by depleted cognitive control through heightened emotional sensitivity (Cracco et al., 2017), it may be that individuals higher in trait aggression have a higher resource deficit to recoup through sleep, which is easily repaired via a healthy amount of sleep (i.e., 8.5 h). Given that aggression and sleep are understudied topics, more studies are required to confirm whether long sleep may be a protective factor for vulnerable adolescents possessing higher trait aggression.

The present study found no evidence to support a relationship between self-reported adolescent sleep quality and aggressive behaviour. This finding is in opposition to the extant literature, which suggests an even stronger relationship for sleep quality and aggression (Van Veen et al., 2021), as compared to sleep duration and aggression (Van Veen et al., 2022). Although the two self-report measures capturing the index of sleep quality (quality of sleep and refreshment) were strongly interrelated (*r* = 0.73), they showed no relationship with measured sleep duration. This finding is consistent with other studies that have found poor agreement between subjective sleep quality and objectively measured sleep (e.g., Girschik et al., 2012). This may indicate that individuals are poor at appraising their own state of refreshment in the morning, or that self-report measures for sleep quality are often too narrow to accurately capture an index of sleep that aligns with objective measures (Girschik et al., 2012). Self-report measures of mood, acting as proxies for aggression, may also be prone to demand effects, whereby participants infer the intention of the study (Mummolo and Peterson, 2019), especially when questions are presented together (e.g., sleep quality and anger). As the present study is the first known study to test sleep quality in relation to aggression using objective behavioural aggression in a controlled environment, the results found here may reduce confidence in previous findings (e.g., Van Veen et al., 2021). Future studies investigating sleep quality could iterate these findings by conducting experimental designs and utilising comprehensive self-report measures of sleep quality, in conjunction with objective measures of behavioural aggression.

### 4.1 Limitations and future directions

As the study did not directly manipulate sleep, we were unable to examine dichotomous extremes. Future experimental studies could manipulate sleep in this way (e.g., 9 h vs. 4 h), ideally using larger and more diverse samples. Any effects would then come more clearly into focus, thereby increasing statistical power and confidence in the robustness and generalizability of the findings of this study. As the study cohort was 100% female adolescents, future studies could also investigate the effects of adolescent male participants or sex differences in adolescent aggression. As male adolescents are known to be more aggressive on average (Buss and Perry, 1992), the pattern of results may be stronger or display a different tendency. Additionally, this study covered a single night, whereas future studies might investigate the cumulative effects of sleep restriction on aggression over several nights.

### 4.2 Conclusion

The present study provides supporting evidence for sleep duration as a moderator in the relationship between trait aggression and aggressive behaviour in adolescents. Additionally, it lends support to the findings of previous studies that have pointed to sleep duration as an important factor in understanding the antecedents of aggressive behaviour and reinforces stated guidelines for sufficient adolescent sleep. In particular, those adolescents who are vulnerable to aggressive behaviour (i.e., high in trait aggression) may benefit the most from attaining adequate sleep in terms of avoiding aggressive behaviour. These findings may help inform future sleep-based interventions for aggressive adolescents and lend further weight to ongoing research, highlighting the challenges faced by adolescents regarding sleep.

## Data Availability

The raw data supporting the conclusions of this article will be made available by the authors without undue reservation.
