# Supplementary material for: Waking up on the wrong side of the bed: sleep duration moderates the association between adolescent trait aggression and observed aggressive behaviour
Source: Front Psychol. 2026 Mar 10;17:1705874. doi: 10.3389/fpsyg.2026.1705874 (PMC13008865; doi:10.3389/fpsyg.2026.1705874)
Supplement: Supplementary file 1 [file Table_1.docx]

Supplementary Material

# Supplementary Figures and Tables

**Table S1**

*Means and Standard Deviations for BPAQ Subscales with Buss and Perry’s (1992) Original Validation Means and Standard Deviations for Females*

| Variable (Range) | *M* | *SD* | *M* (Buss and Perry, 1992) | *SD* (Buss & Perry, 1992) |
| --- | --- | --- | --- | --- |
| BPAQ physical (9 – 30) | 17.75 | 4.71 | 17.90 | 6.60 |
| BPAQ verbal (10 – 24) | 15.28 | 3.56 | 13.50 | 3.90 |
| BPAQ anger (10 – 30) | 17.28 | 5.13 | 16.70 | 5.80 |
| BPAQ hostility (14 – 32) | 23.41 | 5.27 | 20.20 | 6.30 |
| BPAQ comp (49 – 111) | 73.72 | 13.65 | 68.20 | 17.00 |

*Note. N* = 32. Buss and Perry (1992) *n = 641.* BPAQ comp = BPAQ composite (i.e., total).

**Table S2**

*Correlations of Main Study Variables*

| Variable | 1 | 2 | 3 | 4 | 5 | 6 | 7 | 8 | 9 |
| --- | --- | --- | --- | --- | --- | --- | --- | --- | --- |
| 1. Wasabi | - |  |  |  |  |  |  |  |  |
| 2. Sleep dur | -.55** | - |  |  |  |  |  |  |  |
| 3. Sleep Q | -.11 | .09 | - |  |  |  |  |  |  |
| 4. BPAQ | .33 | -.16 | .03 | - |  |  |  |  |  |
| 5. Stress | .10 | .04 | .29 | .36* | - |  |  |  |  |
| 6. Man ch | -.08 | .19 | -.20 | .01 | .12 | - |  |  |  |
| 7. Sus ch | -.30 | .38* | .13 | -.29 | -.06 | .31 | - |  |  |
| 8. Was pref | .08 | -.19* | -.16 | -.08 | -.43* | .27 | .20 | - |  |
| 9. Was like | .09 | -.06 | -.15 | -.06 | -.55** | -.14 | -.08 | .56** | - |

*Note. N* = 32. Sleep Q = sleep quality composite. Stress = DASS-21 stress subscale. Man ch = manipulation check. Sus ch = suspicion check. Was pref = self-reported typical serving preference for wasabi. Was like = self-reported like of wasabi taste. **p* <.05. ***p* < .01.
